# Patient‐reported distress and age‐related stress biomarkers among colorectal cancer patients

**DOI:** 10.1002/cam4.3914

**Published:** 2021-05-01

**Authors:** Hyrum S. Eddington, Megan McLeod, Amber W. Trickey, Nicolas Barreto, Katherine Maturen, Arden M. Morris

**Affiliations:** ^1^ S‐SPIRE Center Department of Surgery Stanford University Stanford California USA; ^2^ University of Michigan Medical School Ann Arbor Michigan USA; ^3^ Department of Radiology University of Michigan Medical School Ann Arbor Michigan USA

**Keywords:** biomarker, colorectal cancer, cortisol, distress, patient reported outcomes, sarcopenia

## Abstract

**Objective:**

Distress among cancer patients has been broadly accepted as an important indicator of well‐being but has not been well studied. We investigated patient characteristics associated with high distress levels as well as correlations among measures of patient‐reported distress and “objective” stress‐related biomarkers among colorectal cancer patients.

**Methods:**

In total, 238 patients with colon or rectal cancer completed surveys including the Distress Thermometer, Problem List, and the Hospital Anxiety and Depression Scale. We abstracted demographic and clinical information from patient charts and determined salivary cortisol level and imaging‐based sarcopenia. We evaluated associations between patient characteristics (demographics, clinical factors, and psychosocial and physical measures) and three outcomes (patient‐reported distress, cortisol, and sarcopenia) with Spearman's rank correlations and multivariable linear regression. The potential moderating effect of age was separately investigated by including an interaction term in the regression models.

**Results:**

Patient‐reported distress was associated with gender (median: women 5.0, men 3.0, *p *< 0.001), partnered status (single 5.0, partnered 4.0, *p *= 0.018), and cancer type (rectal 5.0, colon 4.0, *p *= 0.026); these effects varied with patient age. Cortisol level was associated with “emotional problems” (*ρ* = 0.34, *p *= 0.030), anxiety (*ρ* = 0.46, *p *= 0.006), and depression (*ρ* = 0.54, *p *= 0.001) among younger patients. We found no significant associations between patient‐reported distress, salivary cortisol, and sarcopenia.

**Conclusions:**

We found that young, single patients reported high levels of distress compared to other patient groups. Salivary cortisol may have limited value as a cancer‐related stress biomarker among younger patients, based on association with some psychosocial measures. Stress biomarkers may not be more clinically useful than patient‐reported measures in assessing distress among colorectal cancer patients.

## Graphical abstract

Patient‐reported distress is closely associated with psychosocial and physical needs experienced by colorectal cancer patients, while cortisol is limited in its association to certain needs and younger patients. Stress biomarkers may not be as reliable as patient‐reported distress in understanding the colorectal patient experience.

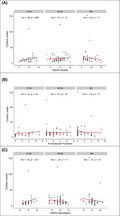

## 1 INTRODUCTION

Cancer‐related distress, defined as “a multifactorial unpleasant experience of a psychological, social, and/or spiritual nature that may interfere with the ability to cope effectively with cancer, its physical symptoms, and its treatment”,^1^ has been termed the “sixth vital sign” due to its prevalence and association with adverse clinical outcomes.^2^ In fact, the American College of Surgeons Commission on Cancer now requires documentation of distress as part of Comprehensive Cancer Center accreditation.^3^ Previous literature suggests that cancer patients manifest measurable physiologic stress as well,^4^, ^5^ and the relationship between physiologic stress and patient‐reported distress is an active area of research.^6^, ^7^, ^8^

Causal mechanisms to explain the associations between patient‐reported distress, physiologic stress, and clinical outcomes are incomplete, however, and require further study to provide a framework to improve patient care. Identification of patient characteristics associated with or even predictive of high levels of distress could clarify the nature of distress in cancer patients and guide development of appropriate interventions to minimize distress‐related adverse outcomes. Benefits of understanding and ameliorating distress among patients with cancer may apply not only to patients, but to healthcare systems as well, as distressed patients often experience longer hospital stays and incur higher healthcare costs.^9^, ^10^, ^11^

Previous studies of the relationship between distress and poor outcomes among cancer patients have been limited to mixed cancer populations and may miss concerns related to cancer sub‐types.^12^ Although distress levels in colorectal cancer patients have been examined previously,^13^, ^14^, ^15^ the relationship that distress has with other aspects of the colorectal cancer patient experience (e.g., psychosocial, physical challenges) has not been explored, despite the fact that colorectal cancer is the third most common and second most lethal cancer in the United States^16^ and is associated with specific socially stigmatized challenges.^17^

In this study, we examined relationships between patient‐reported distress and two physiologic biomarkers of stress: salivary cortisol and sarcopenia. Additionally, we analyzed the associations of each indicator with demographic, clinical, psychosocial, and physical variables. We focused on two questions:

1. How are demographic, clinical, psychosocial, and physical factors associated with higher levels of patient‐reported distress and physiologic stress?
2. Are subjective measures of patient‐reported distress correlated with objective measures of physiologic stress?

## 2 METHODS

### 2.1 Study population

After study approval by the University of Michigan Institutional Review Board, we approached sequential patients referred for consultation at a tertiary multidisciplinary colon and rectal cancer (CRC) clinic^18^ over a 2‐year period and invited them to participate in the current study. Simultaneously, we created a prospective clinical registry of patients seen at the multidisciplinary clinic including information from in‐person surveys and chart review. Data were abstracted by research assistants and validated by clinician members of the research team. The clinical registry was reviewed regularly to ensure that each patient's record was updated until they completed treatment and had their first 3‐month surveillance visit, at which time the record was designated as complete. For the current study, patients were included if they had a new diagnosis of colon or rectal adenocarcinoma, were able to read, write, and speak English, and provided informed consent. Patients with other diagnoses such as anal squamous cell carcinoma, gastrointestinal stromal tumors (GIST), carcinoid, melanoma, or appendiceal cancer were excluded from the study. Patients who were prescribed medications that affect salivary cortisol levels (i.e., estrogens, synthetic glucocorticoids, androgens, and phenytoin) were also excluded for analyses involving cortisol as a distress indicator.

### 2.2 Clinical and psychosocial measures

Demographic and clinical data for each eligible patient were abstracted from the electronic medical record. Abstracted demographic data included age, sex, race, and primary insurance (Medicare, Medicaid, Other, or None/Self‐Pay). Abstracted clinical data include cancer type and cancer stage.

Patient‐reported distress and psychosocial variables including social, emotional, and physical needs were collected via a survey administered during the first clinical visit. Patient‐reported distress was assessed using the National Comprehensive Cancer Network (NCCN) Distress Thermometer^19^ and Impact Thermometer^20^ (collectively DIT). Psychosocial variables were assessed using the NCCN Problem List^21^ and the Hospital Anxiety and Depression Scale (HADS).^22^ The DIT includes two measures which patients rate on a 1–10 scale: first, the distress they are experiencing, and second, the impact that distress has on their day‐to‐day life. The Problem List allows patients to indicate unmet needs contributing to their distress, classified as “emotional,” “physical,” “spiritual,” “social,” and “practical” problems. Measures for each of these variables were created by summing the total number of problems a patient indicated in each category. We created an additional measure, total problems, by summing all problems indicated by the patient. For the purpose of this study, we supplemented the standard Problem List with additional problems relevant to CRC patients: stoma bag, flatulence, and strength, which refers to the patient's perception of strength or vitality as opposed to weakness. The previously validated HADS instrument can be used to classify each patient as normal (0–7), borderline,^8^, ^9^, ^10^ or abnormal^11^, ^12^, ^13^, ^14^, ^15^ for separate domains of depression and anxiety; however, we used the continuous scale of the instrument in our statistical analysis [12].

### 2.3 Salivary cortisol

Eligible patients were provided with a saliva collection kit (Sarstedt Inc., Nümbrecht, Germany). Patients received instructions to chew a cotton roll at 3:00 PM any day during the week following their appointment, and received one reminder phone call. Given the diurnal variation in cortisol levels, 3:00 PM was chosen because it has the highest likelihood of producing an unaffected, undistorted cortisol measurement.^23^ Saliva samples were mailed in the accompanying envelope for laboratory assessment of cortisol content.

### 2.4 Sarcopenia

Patient frailty was assessed by psoas density abstracted from computed tomography (CT) scans collected as part of the initial clinical evaluation. We calculated two morphometric indicators of sarcopenia, total psoas muscle area and mean psoas muscle density,^24^ from CT scans using algorithms programmed in the University of Michigan Analytic Morphomics Lab.^25^, ^26^ Previous literature has demonstrated these measures’ ability to diagnose sarcopenia as well as their association with adverse patient outcomes.^27^, ^28^

Patient CT images in closest temporal proximity to date of study consent (up to 60 days) were loaded. CT imaging technique including dose parameters and contrast administration varied with patient and institution, but 5 mm sections were used for all study measurements. At the level of the superior endplate of L4, bilateral psoas muscles were manually contoured and the sum of their areas recorded. This value was normalized by patient height for subsequent comparisons. Regions of interest were created and CT density (HU with SD) recorded including full manual contour of bilateral psoas muscles as above. All measurements were performed by a single abdominal radiologist with 7 years of post‐residency experience on a dedicated workstation (GE Advantage Workstation, v. 4.6, Waukesha, WI).

### 2.5 Statistical analysis

The primary study outcome was patient‐reported distress (1–10 scale), and the two secondary outcomes were two biomarkers: salivary cortisol (ng/mL) and sarcopenia (measured as total and mean psoas density). To test for associations between these outcomes and patient characteristics (i.e., gender, race, partnered status, cancer type, and cancer stage), we performed Wilcoxon rank‐sum tests. In addition to patient characteristics, we evaluated the relationships of patient‐reported distress, cortisol, and sarcopenia with HADS anxiety, HADS depression, and Problem List measures (i.e., counts of emotional, social, physical, practical, and total problems) using Spearman's rank correlations. We then performed multivariable linear regression to estimate effects on continuous outcomes: patient‐reported distress and salivary cortisol concentration. In order to control for outliers, concentrations of salivary cortisol were log‐transformed prior to model creation. Separate models were created for each potential predictor variable of interest (i.e., anxiety, depression, “emotional problems,” “social problems,” “practical problems,” “physical problems,” and total problems) controlling for patient age, sex, race, partnered status, cancer type, and cancer stage.

We further investigated whether patient age acted as a modifier of the relationships between patient measures with patient‐reported distress and salivary cortisol. To evaluate this potential moderation effect, analyses for each variable set were performed for the entire study population and then stratified by age categories: 15–49 years, 50–65 years, and 66+ years. After stratified analyses, interaction terms between age and the primary independent variable were added to the multivariable models for anxiety, depression, and “emotional” problems for which these terms were significant. Statistical significance was assessed at the level of *p *< 0.05. Statistical analysis was performed using R software (R Foundation for Statistical Computing, Vienna, Austria). ^29^

## 3 RESULTS

### 3.1 Patient characteristics

We approached 315 patients for participation in the study; 268 (85%) consented to participate and 238 (76%) completed both the survey and the salivary cortisol samples. Among 238 participants, 59% were male, 86% were White (Table 1). Twenty‐one percent were ≤49 years, 44% were 50–65 years, and 35% were ≥66 years. Thirty‐seven percent of participants reported Medicare as their primary insurance, 8% reported Medicaid, and 54% reported “Other.” Sixty‐three percent of participants were diagnosed with colon cancer, while 37% were diagnosed with rectal cancer. Cancer stage was obtained from the electronic health record and included 9% Stage I, 16% Stage II, 39% Stage III, and 36% Stage IV.

**TABLE 1:** Demographic and clinical characteristics of the patient cohort

Factor	Overall, n=238	Age Group (years)		
		15–49, n=50	50–65, n=104	66+, n=84
Sex				
Male	59%	50%	59%	64%
Female	41%	50%	41%	36%
Race				
White	86%	80%	85%	91%
Non‐White	14%	20%	15%	9.5%
Relationship Status				
Single	32%	28%	24%	43%
Partnered	69%	72%	76%	57%
Insurance				
Medicare	37%	6.0%	11%	89
Medicaid	8.0%	8.0%	14%	0%
None/Self‐Pay	.40%	2.0%	0%	0%
Other	54%	84%	75%	11%
Cancer Type				
Colon	63%	58%	61%	69%
Rectal	37%	42%	39%	31%
Cancer Stage				
I	9.3%	6.0%	6.9%	14%
II	16%	20%	17%	13%
III	39%	36%	41%	38%
IV	36%	38%	35%	35%

### 3.2 Patient characteristics and reported distress

Patient‐reported psychosocial distress was associated with sociodemographic and clinical characteristics (Table 2). Women reported higher levels of distress than men (median: men 3.0, women 5.0, *p *< 0.001), single patients reported more distress than partnered patients (median: single 5.0, partnered 4.0, *p* = 0.018), and patients diagnosed with rectal cancer reported higher distress than those diagnosed with colon cancer (median: colon 4.0, rectal 5.0, *p* = 0.026). When stratified by age group, the effects of gender and partnered status on patient‐reported distress differed between the youngest and oldest age groups. Specifically, higher levels of distress among women relative to men were significant only among patients aged 50 and older (median: age 50–65 F 5.0, M 4.0, *p* = 0.015; age 66+ F 5.0, M: 2.0, *p* = 0.002). Similarly, the differences in median distress by partnered status were observed only among the youngest age group (median distress age <50 years: single 7, partnered 3.5, *p* = 0.005). The overall relative difference in median distress between colon and rectal cancer was consistent among age groups, although the differences were not statistically significant in age‐stratified analyses.

**TABLE 2:** Patient factors and patient‐reported psychosocial distress, stratified by age group. patient factors

Sociodemographic and clinical	Median distress (range 1–10) by age group (years)			
	Overall	15–49	50–65	66+
Sex				
Female	5.0**	5.0	5.0**	5.0**
Male	3.0	4.5	4.0	2.0
Race				
White	4.0	4.0	5.0	4.0
Non‐White	4.5	5.5	3.5	3.0
Relationship status				
Single	5.0**	7.0**	5.0	5.0*
Partnered	4.0	3.5	4.0	3.0
Cancer site				
Colon	4.0**	4.0	4.0*	2.0
Rectal	5.0	6.0	5.0	4.0

HADS/PL Measures	Correlation by Age Group (years)			
	Overall	15–49	50–65	66+
Anxiety	0.55**	0.62**	0.59**	0.44**
Depression	0.19**	0.22**	0.13*	0.22*
Physical Problems	0.42**	0.41**	0.42**	0.43**
Emotional Problems	0.61**	0.57**	0.61**	0.62**
Social Problems	0.28**	0.26**	0.30**	0.27**
Practical Problems	0.22**	0.17*	0.35**	0.02
Total Problems	0.57**	0.54**	0.58**	0.55**

Abbrviations: PL, Problem List; HADS, Hospital Anxiety and Depression Scale.

For categorical demographic variables, the group medians are shown along with the p‐value of the corresponding Mann‐Whitney U test. For psychosocial numeric variables, Spearman rank correlation (Spearman's rho) is reported along with corresponding *p*‐value. *P*‐value keys are as follows:

* *p *< 0.1;

** *p *< 0.05.

### 3.3 Psychosocial measures and distress outcomes

Every patient survey measure (i.e., Problem List measures, HADS anxiety, HADS depression) at every age group was positively correlated with patient‐reported distress with two exceptions. Depression was only correlated with patient‐reported distress in the youngest age group, and “practical problems” was only correlated with patient‐reported distress for patients aged 50–65 (Table 2).

We found no associations between demographics or psychosocial measures with sarcopenia, with the exception of a significant difference in total psoas area between men and women (M: 2998.7, 1832.3, *p* = 0.001). Nor did we find any correlation between patient‐reported distress and either salivary cortisol or sarcopenia.

**FIGURE 1:**
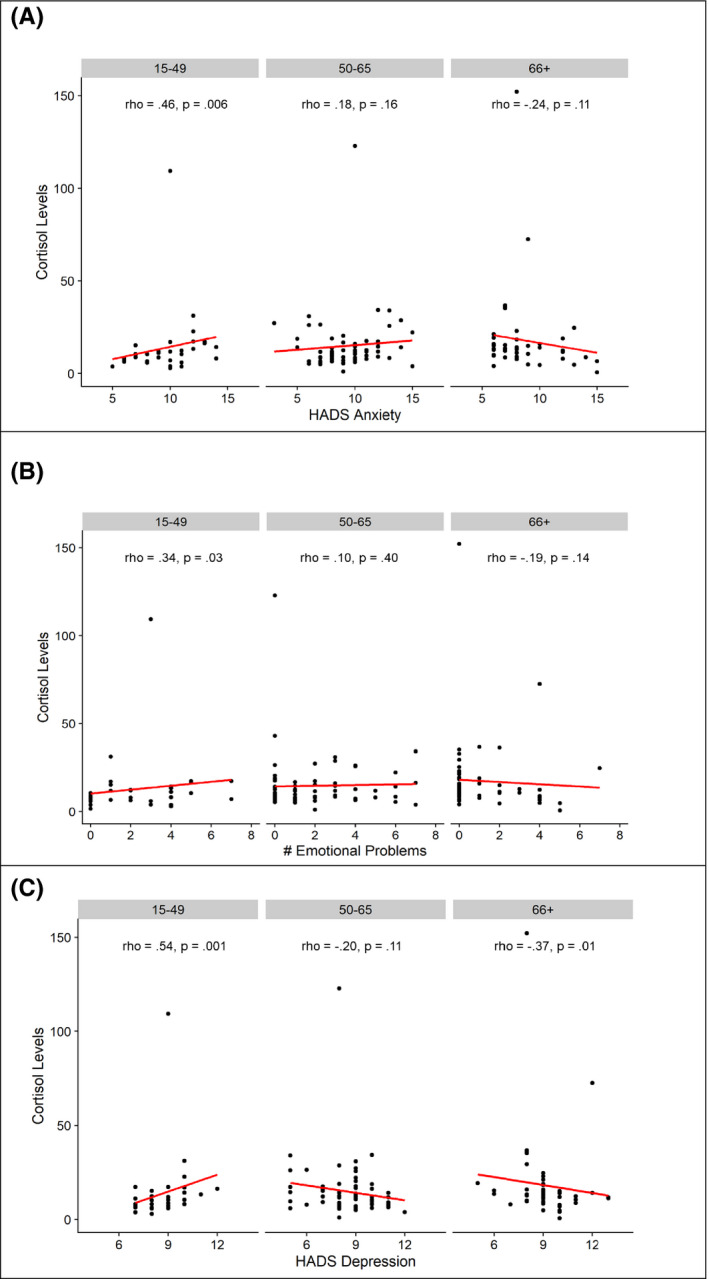
Correlation between cortisol and psychosocial factors: anxiety, depression, and number of unmet emotional needs. Scatterplots fitted with linear models show positive correlations between cortisol and anxiety, depression, and unmet needs in age group 15–49, and between cortisol and emotional needs in age group 50–65. Anxiety is positively correlated with cortisol in the 15–49 and 50–65 age groups. Unmet emotional needs are positively correlated with cortisol in the 15–49 age group. Depression in positively associated with cortisol in the 15–49 age group and negatively correlated with cortisol in the 66+ age group. Test statistics and *p*‐values were calculated using Spearman rank correlations. HADS =Hospital Anxiety and Depression Scale

Within specific age group stratifications, anxiety, depression, “emotional problems,” and “social problems” were correlated with cortisol levels (Table 3). Anxiety and cortisol levels were positively correlated in young patients (15–49: *ρ* = 0.46, *p* = 0.006) but showed no significant pattern in older age groups (Figure 1). Depression was also positively correlated with cortisol in the 15–49 age group (*ρ* = 0.54, *p* = 0.001), but was negatively correlated with cortisol in the oldest 66+ age group (*ρ* = −0.37, *p* = 0.010). Patient‐reported “emotional problems” were significantly correlated with cortisol levels in the youngest age group (age 15–49: *ρ* = 0.34, *p* = 0.030) but no significant correlation with cortisol was observed in older patients. “Social problems” were positively correlated with cortisol only in the 50–65 age group (*ρ*: 0.35, *p* = 0.003) with relatively minimal correlation coefficients in other age groups. “Practical problems” were also positively correlated with cortisol in the 50–65 age group (*ρ*: 0.31, *p* = 0.008) with little correlation in other age groups.

**TABLE 3:** Correlates of cortisol level stratified by age group

Patient factors	Median cortisol (ng/mL) by age group (years)			
Sociodemographic and clinical	Overall	15–49	50–65	66+
Sex				
Female	11	8	11.7	12.1
Male	10.6	10.4	10.1	12.6
Race				
White	11.1	8.6	11.7	12.3
Non‐white	8.2	6.5	7.6	14.9
Relationship status				
Single	11.7	9.1	10.4	12.8
Partnered	10.4	8.6	10.6	11.8
Cancer site				
Colon	10.5	8.1	10.1	12.9
Rectal	11	9.6	11	11.2

HADS/PL Measures	Correlation by Age Group (years)			
	Overall	15–49	50–65	66+
Anxiety	0.08	0.46**	0.18	−0.24
Depression	−0.06	0.54**	−0.20	−.37**
Physical problems	0.13*	0.20	0.14	0.10
Emotional problems	0.03	0.34**	0.10	−0.19
Social problems	0.19**	0.19	0.35**	0.04
Practical problems	0.09	0.02	0.31**	−.05
Total problems	0.13*	0.30*	0.20*	−0.01

Abbreviations: HADS, Hospital Anxiety and Depression Scale; PL, Problem List.

For categorical, demographic variables, the group medians are shown along with the p‐value of the corresponding Mann–Whitney *U*‐test. For psychosocial, numeric variables, Spearman's rank rho is reported along with corresponding *p*‐value. *P*‐value keys are as follows:

* *p *< 0.1;

** *p *< 0.05.

### 3.4 Multivariable regression analysis

Separate multiple regression models of patient‐reported distress with anxiety, depression, “emotional problems,” “social problems,” “physical problems,” “practical problems,” and total problems as primary independent variables and adjustment for age, gender, race, partnered status, and cancer type demonstrated significant associations between cancer type and self‐reported distress for all measures. Gender was associated with patient‐reported distress in all models except for “emotional problems.” Women had higher levels of patient‐reported distress with the largest gender effects in “practical problems” (*B* = 1.39, *p *< 0.001), “social problems” (*B* = 1.32, *p* = 0.007), and “physical problems” (*B* = 1.28, *p <* 0.001). Partnered patients reported significantly lower distress in all measures except for “physical problems” and total problems. The largest partnered status effects were observed in depression (*B *= −1.29, *p *= 0.009), anxiety (*B *= −1.18, *p *= 0.004), and “social problems” (*B *= −1.20, *p *= 0.007). Covariates cancer stage and race had no significant associations with salivary cortisol or patient‐reported distress for any independent variables.

Multiple regression analysis of cortisol levels confirmed the presence of an interaction between age and psychological measures, anxiety, depression, and “emotional problems.” When controlling for patient demographics, cancer type, and cancer stage, the effect of one HADS anxiety unit on cortisol was negative in the 66+ age group compared to the 15–49 age group (*B *= −0.21, *p *= 0.002). Similarly, the correlation between “emotional problems” and salivary cortisol was negative in the 66+ age group relative to the 15–49 age group (*B *= −0.18, *p *= 0.012). For depression, negative interaction effects were observed in both the 50–65 age group (*B *= −0.35, *p *= 0.003)) and the 66+ age group (*B *= −0.38, *p *= 0.002)).

## 4 DISCUSSION

We found that, among patients with colorectal cancer, patient‐reported distress was significantly associated with patient demographic and clinical characteristics and was, with the exception of depression, positively correlated with all HADS and Problem List measures among all age groups. Gender, partnered status, and cancer type but not race were associated with patient‐reported distress. Our findings have implications for determining the most sensitive distress indicators among this patient population.

In our analysis of patient demographics associated with patient‐reported distress, we found that the gap in distress between single and partnered individuals was driven by the large disparity of median distress in the 15–49 age group (S: 7, P: 3.5), suggesting that the distress associated with cancer as a single individual is exacerbated among younger cancer patients. While literature consistently suggests that youth and single status are each separately associated with high distress levels among other cancer populations,^30^, ^31^, ^32^ our study additionally implies that the experience of distress in young and single cancer patients may be even more acute than either of these life circumstances alone.

Our finding that median distress was higher among women is consistent with the breast cancer literature,^33^, ^34^ which supports a hypothesis that women report more distress related to body image and other social effects.^35^, ^36^ However, we recognize the contrasting findings of other colorectal cancer patient literature which report higher distress levels for male patients.^37^, ^38^ We do note in these cases that the distress scales used are much more exhaustive and focused around specific psychological disorders rather than a single metric of overall distress, suggesting that these differences in results most likely reflect variation in how the distress is measured rather than a lack of validity. The gender difference in median distress was largest in the oldest 66+ age group. Women also had lower median psoas density, which would normally indicate higher levels of sarcopenia but may have been due to innate gender differences in muscle composition.^39^ A slight increase in median distress was observed among patients with rectal vs. colon cancer. These findings are consistent with previous literature indicating distress related to radical surgery, body image, social stigma, and the bother of caring for a potentially permanent stoma.^40^, ^41^

The finding that salivary cortisol was only positively correlated with depression, anxiety, and emotional problems among younger age groups suggests that cortisol may be an ineffective biomarker of cancer‐related distress for older patients. Age results in decreased activity within the hypothalamic–pituitary–adrenal (HPA) axis.^42^ This decrease in sensitivity may explain why anxiety and emotional problems show no correlation with salivary cortisol in the 66+ age group. Notably, depression is negatively correlated with salivary cortisol in the 66+ age group (Table 3, Figure 1B), which seems to be inconsistent with an HPA axis reduction explanation. However, recent literature suggests that high cortisol levels in elderly individuals along with reduced axis sensitivity are associated with adverse neurological outcomes including depression.^42^ Why this pattern is only seen in depression and not in anxiety in our study is currently unclear.

We found no correlation between patient‐reported distress and sarcopenia or salivary cortisol, but did find a significant positive correlation between HADS and Problem List measures, suggesting that biomarkers are not superior to patient‐reported measures for distress. Similar to salivary cortisol, sarcopenia may be influenced by confounding variables such as age. Additionally, there were no significant pairwise correlations among the three distress measures, suggesting that these three measures are not equivalent in assessing patient distress, though biomarkers may demonstrate usefulness in determining likelihood of other clinical outcomes.

Our study was subject to several limitations. For analytical purposes we collapsed all non‐white race categories into one variable to optimize statistical power. In addition, we noted 4 subjects with high outlier cortisol levels. Although we repeated testing to confirm these results and searched for exogenous sources, it is possible that these data resulted from undocumented medications such as Selective Serotonin Reuptake Inhibitors (SSRI’s).^43^ While we excluded patients on medications that may affect cortisol levels, the remaining presence of a small number of extreme outliers suggests some relevant medications might not have been captured. We expect that future studies with additional medication exclusion parameters would find similar results, and we recommend a more diverse sample population in order to draw race‐related conclusions. Additionally, future studies should consider collecting information about family history of CRC to examine its effect on psychosocial distress. We also recognize that our sample has a high percentage of young patients (65% <65 years of age and), which could raise concerns regarding generalizability. We note that, accordingly, we stratified our analyses by age which resulted in the significant and potentially impactful results regarding young patients noted in this discussion. We also note an ongoing and statistically significant trend toward younger age at diagnosis^44^, ^45^ and our population‐based survey findings in which approximately 53% of respondents with Stage III colorectal cancer were younger than age 65,^46^ which taken together suggest a shift towards a younger average colorectal patient age.

Our study found that gender, age, partnered status, and cancer type are all important patient characteristics to consider when evaluating a patient's risk for psychosocial distress. Additionally, our study found an overall lack of concordance between patient‐reported psychosocial distress and biomarkers of physiologic stress. While it might be expected that objective biomarkers are less biased therefore more reliable indicators of patient distress, we found few associations between cortisol or sarcopenia and patient characteristics, HADS and Problem List measures. In conclusion, our data suggest that given the difficulty of reliably measuring patient distress from biomarkers, patient‐reported psychosocial distress measures are more useful than biomarkers for clinicians to understand and respond to the cancer patient experience.

## CONFLICTS OF INTEREST

We have no conflicts of interest to disclose.

## AUTHOR CONTRIBUTIONS

AMM and MM designed the experiment and collected the relevant data. KM performed radiological measurements for relevant variables. HE performed the statistical analysis. AT and NB verified the statistical methods. HE wrote the manuscript, and all co‐authors contributed to data interpretation and critical revision of the work.

## PRECIS

Patient‐reported distress is closely associated with psychosocial and physical needs experienced by colorectal cancer patients, while cortisol is limited in its association to certain needs and younger patients. Biomarkers of distress may not be as reliable as patient‐reported distress in understanding the colorectal patient experience.

## ETHICS APPROVAL

After study approval by the University of Michigan Institutional Review Board, we approached sequential patients and invited them to participate in the current study. Patients were included if they had a new diagnosis of colon or rectal adenocarcinoma, were able to read, write, and speak English, and provided informed consent.
